# Identification and validation of an explainable prediction model of favorable outcome under integrative medicine treatment exposure in DKD adult patients: a retrospective cohort study

**DOI:** 10.3389/fdgth.2026.1803468

**Published:** 2026-07-16

**Authors:** Li Jiang, Haojun Zhang, Yanmei Wang, Meihua Yan, Xiai Wu

**Affiliations:** 1 Diabetes Department of Integrative Medicine, China National Center for Integrated Traditional Chinese and Western Medicine, China-Japan Friendship Hospital, Beijing, China; 2 Institute of Clinical Medical Sciences, China-Japan Friendship Hospital, Beijing, China

**Keywords:** diabetic kidney disease, integrative medicine treatment, machine learning (ML), prediction model, retrospective cohort study

## Abstract

**Background:**

Diabetic kidney disease (DKD) shows heterogeneous responses to integrative medicine treatment (IMT). A critical unmet need in DKD management is the inability to predict IMT response, which is essential for advancing personalized treatment strategies.

**Aim:**

To develop and validate an explainable model for predicting likelihood of favorable outcome under IMT exposure in adult DKD patients.

**Methods:**

A retrospective cohort comprising 7,400 patients with diabetic kidney disease (DKD) from 2010 to 2018 was analyzed. Among them, 3,900 consecutive cases diagnosed between 2010 and 2014 were randomly divided in a 7:3 ratio into a training set (*n* = 2,730) and an internal test set (*n* = 1,170), while 3,500 cases from 2014 to 2018 served as an temporal validation cohort. Feature selection was performed using the Boruta algorithm, followed by LASSO regression, Random Forest, and XGBoost-SHAP analysis. Predictive models including logistic regression, LASSO, and XGBoost were developed and evaluated based on the area under the receiver operating characteristic curve (AUC). Further assessment of model performance included sensitivity, specificity, positive predictive value (PPV), negative predictive value (NPV), accuracy, and the F1 score. The optimal XGBoost classifier was subsequently deployed as an interactive, single-page web application using the R Shiny framework.

**Results:**

XGBoost performed best (AUC = 0.783 in training, 0.715 in test and 0.762 in validation set), with 10 key variables, namely creatinine (cr), uric acid (ua), age, red blood cell count (rbc), urea, glucose (glu), platelet count (plt), calcium (ca), white blood cell count (wbc), and sodium (Na). The web app enabled real-time prediction (https://predictionfordkd.shinyapps.io/Prediction/).

**Conclusion:**

The model effectively predicts likelihood of favorable outcome under IMT exposure in DKD, aiding personalized treatment.

## Graphical Abstract

**Figure:**
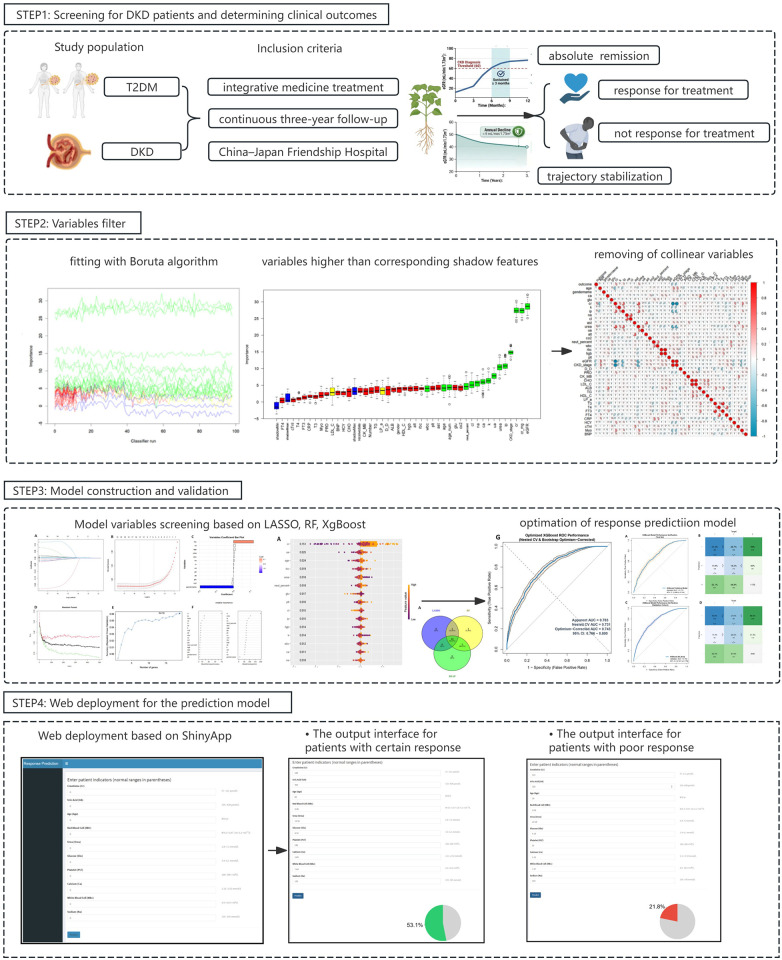

## Introduction

Diabetic kidney disease (DKD) now affects 30%–40% of the 537 million adults living with diabetes (1) and has become the leading cause of end-stage kidney disease worldwide (2). Despite intensified glycaemic, blood-pressure and lipid control, the age-standardised incidence of DKD continues to rise by 1.5% per year (3), driven by population ageing and the obesity epidemic. Landmark trials with SGLT2 inhibitors, non-steroidal mineralocorticoid receptor antagonists and GLP-1 receptor agonists have recently retarded eGFR decline (4, 5); however, residual albuminuria persists in partial treated patients, and 15%–20% still progress to dialysis within a decade (6). These figures underscore an unresolved bottleneck: the absence of tools that prospectively identify individuals who will derive meaningful renal protection from any given therapy.

Over the past decade, integrative medicine treatment (IMT)-defined here as guideline-based conventional therapy plus standardised Chinese medicine protocols-has shown incremental efficacy in DKD. Meta-analyses of 20 randomised trials (*n* = 2,719) reported that integrative medicine treatment reduced albuminuria [SMD −0.56, 95% CI (−1.04 to −0.08), *I*^2^ = 64%, *p* = 0.002] and increased the eGFR significantly [MD 6.28 mL/min; 95% CI (2.42 to 10.14), *I*^2^ = 0%, *p* = 0.001] in DKD patients compared with the conventional treatment (7). Another meta-analysis revealed that compared to the sole conventional treatment, the use of IMT after 24 weeks showed significant improvement in the experimental group over the control group, with UAER [MD = −15.94 (95% CI: −30.67–1.22); *P* = 0.03] and 24 h UP [MD = −0.20(95% CI:−0.36–0.05);*P* = 0.01] (8). Mechanistic studies attributed these gains to multi-pathway modulation of TGF-β/Smad, AGE-RAGE and NLRP3 inflammasome signalling (9). Consequently, IMT has been incorporated into the Chinese Society of Nephrology consensus (2025) as a recommended option for stage G1–G3 DKD (10).

**Figure 1:**
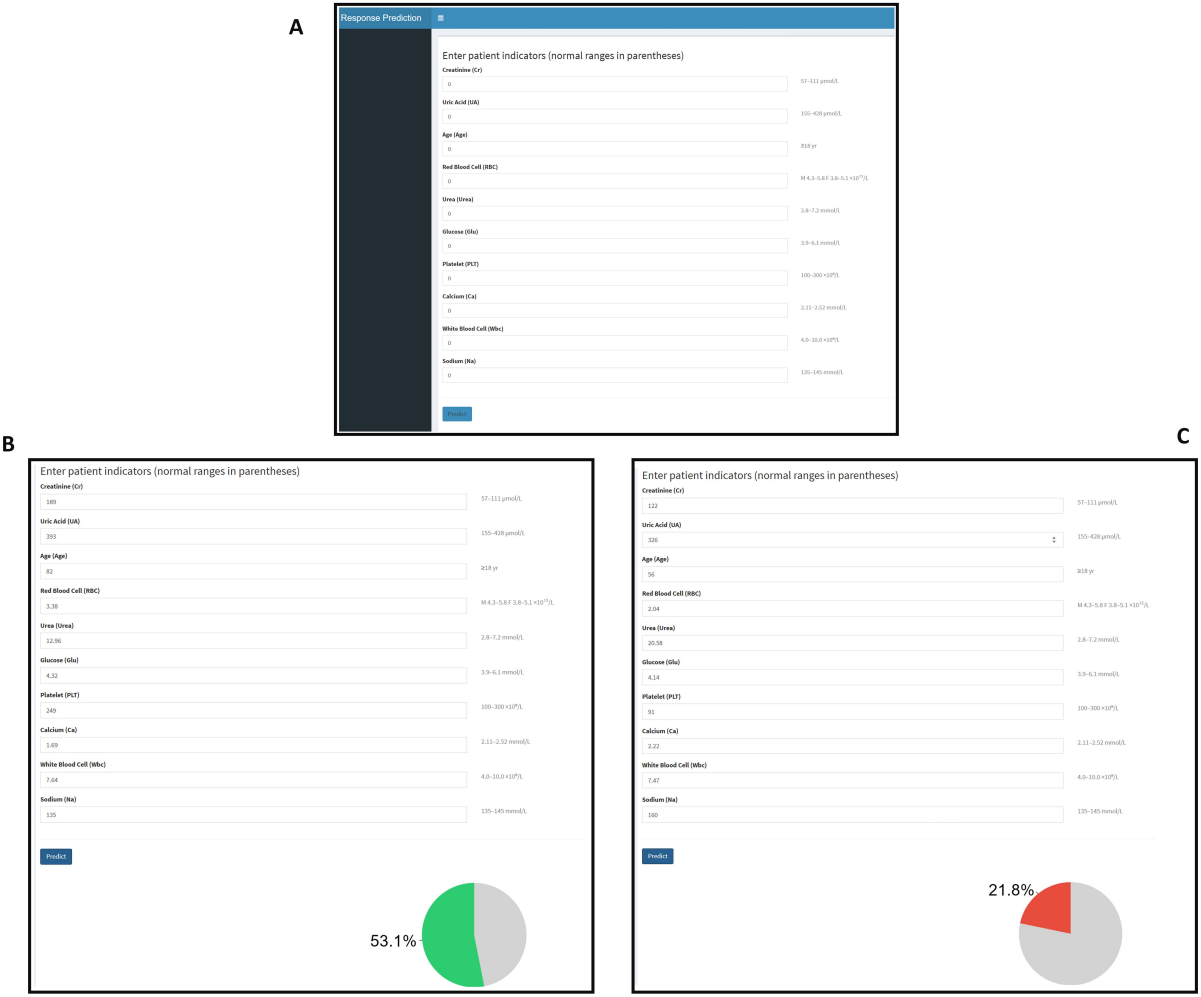
Web deployment for the prediction model. **(A)** The UI interface for variable input, where various patient indicators with their normal ranges in parentheses can be entered. **(B)** The output interface for patients with certain response, showing a 53.1% probability of favorable response via a pie chart. **(C)** The output interface for patients with poor response, presenting a 21.8% probability of unfavorable response through a pie chart.

Yet clinical adoption of IMT was constrained by pronounced inter-individual heterogeneity. For example, IMT significantly improved hormone levels in diminished ovarian reserve patients, particularly in younger, nulliparous women with moderate ovarian reserve (11). In real-world clinical practice, IMT can effectively reduce the body weight of obese patients. For patients with severe obesity, the weight loss effect is better in females than in males (12). The treatment responses in the field of chronic kidney disease were also similar. This variability necessitated data-driven stratification to fulfil the promise of precision nephrology. In the landscape of chronic kidney disease (CKD) management, predictive models have emerged as pivotal tools to address the inherent heterogeneity in therapeutic responses (13, 14). Yet their application in guiding IMT for DKD remained relatively nascent. Existing predictive models for DKD progression primarily focus on conventional risk factors such as age, gender, baseline eGFR, albuminuria, and glycemic control, utilizing linear regression or simple machine learning (ML) algorithms like logistic regression (15–17). For instance, the CKD-EPI equation, a widely used conventional model, estimates eGFR decline but fails to capture the non-linear, multi-factorial interactions that influence treatment-specific responses (18). More recent ML-based models, such as random forests and gradient-boosted decision trees, have shown improved performance in predicting DKD progression compared to traditional methods, with areas under the receiver operating characteristic curve (AUC) ranging from 0.755 to 0.811 (19, 20). However, these models are predominantly designed to forecast disease outcomes rather than therapeutic efficacy, limiting their utility in clinical decision-making for treatment selection. Machine-learning algorithms excel at capturing non-linear interactions among high-dimensional clinical, omic and therapeutic variables and have repeatedly outperformed conventional regression in therapeutic-response prediction (21). Leveraging a ten-year, single-centre retrospective cohort of adult DKD patients treated at China-Japan Friendship Hospital, we aimed to identify the clinical signature of IMT responders, and develop and internally validate an explainable prediction model that quantifies individual probability of renal benefit from IMT. To bridge the gap between heterogeneous IMT efficacy and bedside decision-making, we sought to develop an openly accessible, explainable prediction tool. Figure 1 offers a preview of the final product: a single-page, interactive web application that instantly estimates the likelihood of favorable outcome under IMT exposure after a clinician enters ten routinely available laboratory values. The following sections describe the retrospective cohort, multi-algorithm feature selection, model validation, and the technical deployment of this bedside aid. the application is served through Shiny Server and becomes accessible via any modern browser at https://predictionfordkd.shinyapps.io/Prediction/. We have provided the normal range for each indicator (Figure 1A). When the response probability exceeds 50%, the result was displayed in green (Figure 1B), and when it was less than 50%, it is represented in red (Figure 1C).

## Methods

### Study population and data source

Between January 2010 and December 2018, we retrospectively screened electronic health records (EHRs) from the Diabetes Department of integrative medicine at China-Japan Friendship Hospital to identify hospitalized patients with type 2 diabetes mellitus (T2DM) and concomitant diabetic kidney disease (DKD). The selection of this study period was based on a preliminary assessment of data availability and completeness. An initial audit of annual discharge records and data integrity indicated that the 2010–2018 timeframe would provide a sufficient number of eligible patients with the required three-year follow-up, thereby meeting the sample size requirements for robust model development and validation.

Prior to patient selection, a formal sample size calculation was performed to ensure adequate statistical power for developing the prediction model. The calculation followed the approach recommended by Riley et al. for multivariable prediction models, implemented using the “pmsampsize” package in R (version 4.3) (22, 23). Parameters were specified as follows: an anticipated Cox-Snell R^2^ of 0.15 based on prior pilot data and literature on DKD progression; an expected outcome (remission) rate of 0.35; an assumed 10 candidate predictors after feature selection; and a target shrinkage factor of 0.9 to maintain model calibration. The calculation indicated that a minimum of 2,850 participants were required in the training set. Accounting for potential missing data and loss to follow-up, the target sample size was increased by 20%, yielding a final requirement of approximately 3,420 patients in the training cohort.

T2DM was ascertained by fasting plasma glucose, glycated hemoglobin (HbA1c), or a 75 g oral glucose tolerance test (OGTT) (24). Estimated glomerular filtration rate (eGFR) was calculated with the CKD-EPI equation using serum creatinine. Eligible adults were aged 18–75 years. The integrative medicine treatment required ≥7 consecutive days of oral Chinese patent medicine or decoction during hospitalization and ≥6 cumulative months of outpatient Chinese medication per year thereafter. The included patients should have a continuous three-year follow-up record. DKD was defined by any of the following confirmed on two of three occasions within 3–6 months: (1) random urinary albumin-to-creatinine ratio (ACR) ≥ 30 mg/g or albumin excretion rate ≥30 mg/24 h; (2) eGFR <60 mL·min^−1^·1.73 m^−2^ persisting >3 months; (3) renal biopsy consistent with diabetic nephropathy (25). Exclusion criteria comprised other primary renal disorders, malignancy, active infection, systemic autoimmune disease, severe cardiovascular or cerebrovascular events, malnutrition, or corticosteroid use within six months preceding enrolment. After deduplication, 444,095 encounters were reduced to 3,900 (2010–2014) and 3,500 (2014–2018) unique patients. The 2010–2014 cohort (*n* = 3,900) exceeded the calculated sample, whereas the 2014–2018 cohort (*n* = 3,500) served as an temporal validation cohort.

### Ethics approval and consent to participate

This study was a retrospective analysis of anonymized EHR extracted from the China-Japan Friendship Hospital (CJFH) Clinical Data Warehouse. The protocol was reviewed and approved by the CJFH Institutional Review Board (approval No. 2025-KY-205-1). Because all data were de-identified before the analyses and no additional intervention or contact with patients was required, the IRB waived the requirement for individual informed consent. The waiver was granted on the following grounds: (1) the research posed no more than minimal risk to the subjects; (2) the waiver would not adversely affect the rights and welfare of the subjects; (3) the study could not practicably be carried out without the waiver; and (4) whenever appropriate, subjects would be provided with additional pertinent information after participation. All electronic records were stripped of direct identifiers (name, ID number, address, telephone, etc.) and replaced by a unique project code. Access to the code list was restricted to two senior data managers behind the hospital firewall; the analytical team received only the anonymized dataset. The database was stored on an encrypted server with password-protected access logs, and all analyses were conducted within the CJFH secure research environment. The study was conducted in accordance with the principles of the Declaration of Helsinki (as revised in 2013) and the local laws and regulations of the People's Republic of China.

### Methods of biochemical analysis

All biochemical indicators were measured using venous blood samples collected after 8–12 h of fasting, except for 24 h urinary protein (24 h PRO) which was determined from 24 h urine collections. Serum electrolytes (Ca, K, Na, Cl, P), glucose (Glu), creatinine (Cr), urea, uric acid (UA), total cholesterol (CHO), triglycerides (TG), high-density lipoprotein cholesterol (HDL-C), low-density lipoprotein cholesterol (LDL-C), albumin (ALB), aspartate aminotransferase (AST), alanine aminotransferase (ALT), and CO_2_ were analyzed by Beckman Coulter AU5800 automatic biochemical analyzer with matching reagents (Beckman Coulter, USA). Complete blood count parameters (WBC, RBC, HGB, PLT, Neut_percent) were detected using SYSMEX XN-9000 hematology analyzer (Sysmex, Japan). Cardiac markers (CK-MB, cTnI, Myo, BNP), thyroid function indicators (T3, T4, FT3, FT4), lipoprotein(a) [LP(a)], C-reactive protein (CRP), homocysteine (HCY), and D-dimer (D-D) were measured via chemiluminescence immunoassay on Architect i2000SR analyzer (Abbott Laboratories, USA) with commercially available kits. 24 h PRO was quantified by pyrogallol red-molybdate method using a specific assay kit (Beijing Solarbio Science & Technology Co., Ltd., China).

### Clinical outcomes

Primary endpoint was remission of DKD following integrative medicine therapy, adjudicated after three consecutive follow-up visits. Remission was satisfied if any of the following criteria were met: (1) Absolute functional remission: sustained recovery of eGFR to >60 mL·min^−1^·1.73 m^−2^ for at least 3 months, coupled with urinary albumin-to-creatinine ratio ≤30 mg/g in at least two of three spot urine samples collected over 3–6 months; (2) Trajectory stabilization: annual eGFR decline <5 mL·min^−1^·1.73 m^−2^, or the post-exposure annual eGFR slope demonstrated a ≥30% slope improvement relative to the individualized pre-exposure historical trajectory (26). Participants fulfilling these remission criteria were classified as Responders; all others were designated Non-responders.

### Statistical analysis

We applied list-wise deletion to remove records with ≥ 80% missing data, and excluded variables whose missingness exceeded 10% to remove low-information predictors. For the remaining incomplete variables, multivariate imputation by chained equations (MICE) was performed with the R package “mice”. The multi-level imputation procedure was executed strictly within the training framework after the initial dataset partitioning. Descriptive statistics are presented as mean ± SD for continuous variables and as counts (percentages) for binary variables; normality was evaluated with the Shapiro–Wilk test. Group comparisons across DKD stages G1–G5 employed the independent-sample *t* test, Mann–Whitney *U* test, or *χ*^2^ test, as appropriate. Baseline associations were quantified with Spearman rank correlations and displayed as a correlation matrix (27). All analyses were conducted in SAS 9.4 (SAS Institute, Cary, NC, USA) and R 4.3; two-sided *P* < .05 was considered statistically significant.

### Model construction, validation, and performance evaluation

A total of 7,400 patients with DKD were enrolled; 3,900 consecutive cases diagnosed between 2010 and 2014 were randomly split 7: 3 into training and internal test sets (28), whereas 3,500 cases presenting from 2014 to 2018 served as an temporal validation cohort. The training set was used to develop a prediction model for integrated Chinese-Western therapy in DKD. The model construction followed a rigorous, multi-stage feature selection process to mitigate overfitting and enhance clinical interpretability. Feature engineering began with the Boruta algorithm implemented in the R package “Boruta” (29). Shadow features were appended to the original variables and 100 iterations were performed; variables whose importance exceeded the binomial-derived threshold were retained, yielding the top 50% candidates. These variables were then subjected to a correlation analysis to identify multicollinearity. In cases of high correlation, clinical relevance and measurement directness were prioritized.

Subsequently, the refined variable set was independently processed by three distinct algorithms: LASSO regression, Random Forest, and XGBoost with SHAP analysis. To ensure algorithmic robustness and rigorous error estimation, nested cross-validation (Nested CV) and bootstrapping were implemented during the model-fitting phase. Within this validation framework, learning curves were constructed by progressively expanding the training scale to dynamically monitor the balance between bias and variance, thereby supporting the robustness of the model. Furthermore, feature stability analysis was performed across iterative validation loops to quantify the selection frequency and constancy of the predictors. The intersection of variables consistently selected by all three methods yielded a final set of ten robust predictors (30).To ensure full reproducibility of our machine learning pipeline, the final optimized hyperparameters obtained via nested cross-validation are comprehensively detailed in Supplementary Table 1.

ROC curves were generated and the area under the curve (AUC) was calculated. Calibration plots and their corresponding reliability estimates (including the Brier score, calibration intercept, and slope) were generated within the training framework utilizing out-of-fold predictions accumulated across the 5-fold cross-validation loops. This approach ensures that the alignment between predicted probabilities and empirically observed outcomes was evaluated on data points functionally unseen during the prospective optimization of individual decision trees, thereby mitigating overfitting bias before expanding to independent testing. Decision-curve analysis (DCA) was applied to compare clinical net benefit. An approximate logistic model with restricted cubic splines (RCS) was then constructed to create a nomogram. SHAP values extracted from XGBoost were summed with the global intercept and transformed via the logistic link to obtain predicted probabilities. The “rms” package was employed to fit an lrm model: key variables were modelled with three-knot RCS, remaining covariates entered linearly, and the nomogram produced a calibrated, interpretable chart.

Model performance was evaluated in the test set using true positive rate (TPR, also known as “sensitivity” and “recall”), true negative rate (TNR, also known as “specificity”), positive predictive value (PPV, also known as “precision”), negative predictive value (NPV), accuracy, as well as F1 score (31). The algorithm exhibiting the highest accuracy was selected. The optimal model was subsequently applied to the temporal validation cohort, ROC analysis repeated, and confusion matrices generated to confirm predictive precision. Compared with the unconstrained boosting setup, our modeling framework represents a revalidated pipeline to mitigate the severe overfitting hazards.

### Sensitivity analysis

To reduced the likelihood of confounding of the optimized XGBoost model, three sensitivity analyses were performed. First, the model was compared against a Baseline Clinical Model (Age + Creatinine + Glucose) using the Area Under the ROC curve (AUC), continuous Net Reclassification Index (NRI), and Integrated Discrimination Improvement (IDI), with 95% CIs estimated via 1,000-fold bootstrapping. Second, a stage-by-stage leave-one-out sensitivity analysis was conducted by sequentially excluding patients from each diabetic kidney disease (DKD) stage (G1 to G5) to ensure the framework identifies robust predictive signatures regarding the likelihood of favorable outcomes rather than baseline disease severity. Third, a stage-stratified analysis was conducted.

### Model web deployment

The trained XGBoost classifier was encapsulated as a single-page, interactive web application using the Shiny framework. Responsive front-end components and server-side logic were built with the R packages shiny and shinydashboard (32). The model object was serialized to RDS format for persistent storage and deserialized at runtime, enabling Model-as-a-Service delivery without retraining. A dynamic form collects patient-level predictors; the back-end invokes the xgboost prediction API to generate real-time probabilities, which are immediately visualized as a color-coded pie chart that communicates risk tiers.

## Results

### Baseline demographics of the DKD patients in the training dataset

A total of 3,900 adult DKD patients were analysed across five DKD stages (Table 1). Mean age rose progressively from 70.5 ± 14.7 years in stage G1 to 77.6 ± 13.5 years in G2 and then declined to 58.6 ± 16.9 years in stage G5. Females constituted 27.1% of G1 but only 48.1% of G5. Serum creatinine, urea, phosphorus and uric acid increased steeply with advancing stage, whereas eGFR, haemoglobin, albumin, RBC count, CO_2_ and calcium declined. Lipid profiles showed higher triglycerides and lower HDL-C in later stages, while LDL-C remained stable. Inflammatory markers (CRP, BNP, HCY) and cardiac biomarkers (cTnI, CK-MB) were markedly elevated in G5 compared with G1. Overall, 52.0% of patients (2029 out of 3,900) achieved therapeutic response. Notably, the response rate increased with the severity of DKD stage, from 17.8% in G1 to 69.3% in G5. Regarding baseline data integrity within the training dataset, missingness was minor and localized exclusively to five hematological parameters (4.18% for neut_percent; 4.00% each for wbc, rbc, hgb, and plt), while all other clinical and biochemical indicators exhibited 100% completeness. To safeguard statistical power, multiple imputation by chained equations (MICE) was executed. The mathematical convergence of the MICE framework was rigorously verified via post-imputation trace plot diagnostics, where the multi-chain Markov trajectories for both the imputed means and standard deviations across consecutive iterations demonstrated stable, stochastic interweaving without any divergent trends, thereby confirming that the imputed values safely preserved the natural clinical variance of the original cohort (Supplementary Figure 1).

**Table 1:** Baseline characteristics stratified by DKD stage in DKD patients from 2010 to 2014.

DKD stage	G5	G4	G3	G2	G1	*p*. overall
Variables, mean (SD)	*N* = 1,572	*N* = 836	*N* = 857	*N* = 281	*N* = 354	
Age	58.6 (16.9)	69.1 (14.6)	77.4 (11.9)	77.6 (13.5)	70.5 (14.7)	<0.001
Gender:						<0.001
Female	756 (48.1%)	343 (41.0%)	229 (26.7%)	79 (28.1%)	96 (27.1%)	
Male	815 (51.9%)	493 (59.0%)	628 (73.3%)	202 (71.9%)	258 (72.9%)	
Ca (mmol/L)	2.15 (0.32)	2.12 (0.27)	2.13 (0.22)	2.15 (0.21)	2.14 (0.19)	0.058
Glu (mmol/L)	5.93 (2.65)	6.66 (3.75)	6.85 (3.29)	7.64 (4.11)	6.93 (3.04)	<0.001
Cr (µmol/L)	720 (281)	303 (68.3)	172 (33.7)	103 (16.2)	68.3 (14.8)	0.000
K (mmol/L)	4.75 (0.83)	4.44 (0.75)	4.30 (0.67)	4.10 (0.59)	3.99 (0.54)	<0.001
P (mmol/L)	1.74 (0.61)	1.35 (0.40)	1.17 (0.37)	1.07 (0.28)	1.02 (0.28)	<0.001
Na (mmol/L)	139 (4.45)	139 (5.71)	140 (5.72)	140 (5.10)	139 (5.66)	<0.001
Cl (mmol/L)	102 (5.77)	105 (6.94)	105 (6.22)	104 (4.94)	103 (5.72)	<0.001
AST (U/L)	32.9 (193)	63.5 (351)	70.4 (311)	42.1 (73.9)	36.4 (50.1)	0.002
Urea (mmol/L)	23.9 (10.1)	18.1 (7.68)	13.6 (6.03)	10.3 (4.94)	7.00 (3.82)	0.000
UA (µmol/L)	426 (152)	447 (159)	441 (143)	371 (123)	294 (117)	<0.001
ALT (U/L)	28.8 (106)	52.5 (293)	53.6 (230)	35.2 (77.0)	34.6 (45.9)	0.006
CO_2_ (mmol/L)	23.2 (4.85)	23.7 (4.81)	24.7 (4.71)	26.8 (4.76)	27.3 (4.54)	<0.001
Neut_percent (%)	69.4 (11.8)	70.5 (13.7)	71.6 (13.5)	71.0 (15.8)	70.0 (14.9)	0.001
WBC (×10^9^/L)	7.30 (4.39)	8.40 (5.43)	8.66 (5.25)	8.55 (4.73)	8.46 (4.41)	<0.001
RBC (×10^12^/L)	3.11 (0.74)	3.25 (0.73)	3.49 (0.75)	3.72 (0.82)	3.80 (0.87)	<0.001
HGB (g/L)	94.9 (22.3)	99.0 (21.4)	107 (22.6)	114 (25.4)	117 (27.4)	<0.001
PLT (×10^9^/L)	182 (80.8)	190 (88.5)	185 (89.5)	184 (81.1)	193 (85.4)	0.082
24 h PRO	1.74 (1.58)	1.58 (1.71)	1.25 (1.53)	1.03 (1.31)	0.45 (0.73)	<0.001
CK-MB (ng/mL)	12.3 (22.6)	15.0 (25.0)	20.8 (68.9)	32.9 (185)	19.8 (47.0)	0.001
cTnI (ng/mL)	0.69 (1.89)	1.30 (4.04)	1.16 (3.82)	1.24 (3.57)	3.72 (7.66)	0.030
Myo (ng/mL)	292 (142)	246 (134)	216 (128)	180 (131)	119 (96.4)	<0.001
BNP (pg/mL)	918 (983)	834 (930)	715 (924)	697 (933)	330 (544)	<0.001
CHO (mmol/L)	4.37 (1.20)	4.34 (1.46)	4.25 (1.75)	4.22 (1.37)	4.10 (1.17)	0.056
LDL-C (mmol/L)	2.50 (0.88)	2.50 (1.08)	2.49 (1.29)	2.43 (0.98)	2.31 (0.82)	0.131
ALB (g/L)	36.3 (6.57)	34.4 (7.31)	34.4 (7.03)	35.8 (6.64)	36.4 (7.35)	<0.001
TG (mmol/L)	1.69 (1.03)	1.67 (1.04)	1.47 (0.99)	1.41 (1.11)	1.34 (1.20)	<0.001
HDL-C (mmol/L)	1.17 (0.37)	1.15 (0.39)	1.09 (0.38)	1.10 (0.38)	1.07 (0.39)	<0.001
LP(a) (mg/L)	234 (273)	239 (278)	189 (259)	141 (218)	122 (169)	<0.001
T3 (nmol/L)	0.78 (0.31)	0.72 (0.24)	0.73 (0.22)	0.67 (0.20)	0.90 (0.22)	<0.001
T4 (nmol/L)	6.21 (2.26)	6.81 (1.99)	6.91 (1.88)	7.21 (2.18)	7.65 (1.73)	<0.001
FT3 (pmol/L)	2.23 (0.79)	2.08 (0.50)	2.23 (0.53)	2.16 (0.48)	2.73 (0.63)	<0.001
FT4 (pmol/L)	0.97 (0.26)	1.11 (0.24)	1.13 (0.24)	1.20 (0.22)	1.19 (0.23)	<0.001
CRP (mg/L)	1.96 (3.44)	2.87 (4.85)	3.46 (5.40)	4.84 (6.76)	5.35 (7.00)	<0.001
HCY (µmol/L)	28.9 (18.4)	24.9 (14.5)	21.5 (11.0)	16.2 (7.37)	14.9 (9.22)	<0.001
D-D (µg/mL FEU)	2.54 (3.07)	2.63 (3.07)	2.83 (3.46)	2.34 (2.98)	2.38 (3.12)	0.169
Response:						<0.001
No	483 (30.7%)	384 (45.9%)	480 (56.0%)	232 (82.6%)	291 (82.2%)	
Yes	1,088 (69.3%)	452 (54.1%)	377 (44.0%)	49 (17.4%)	63 (17.8%)	

AST, aspartate aminotransferase; ALT, alanine aminotransferase; CO_2_, carbon dioxide; Neut_percent, neutrophil percentage; WBC, white blood cell; RBC, red blood cell; HGB, hemoglobin; PLT, platelet; 24 h PRO, 24 h urinary protein; CK-MB, creatine kinase-MB; cTnI, cardiac troponin I; Myo, myoglobin; BNP, B-type natriuretic peptide; CHO, total cholesterol; LDL-C, low-density lipoprotein cholesterol; ALB, albumin; TG, triglyceride; HDL-C, high-density lipoprotein cholesterol; LP(a), lipoprotein(a); T3, triiodothyronine; T4, thyroxine; FT3, free triiodothyronine; FT4, free thyroxine; CRP, C-reactive protein; HCY, homocysteine; D-D, D-dimer.

### Variables associated with favorable outcome under integrative medicine treatment exposure

**Figure 2:**
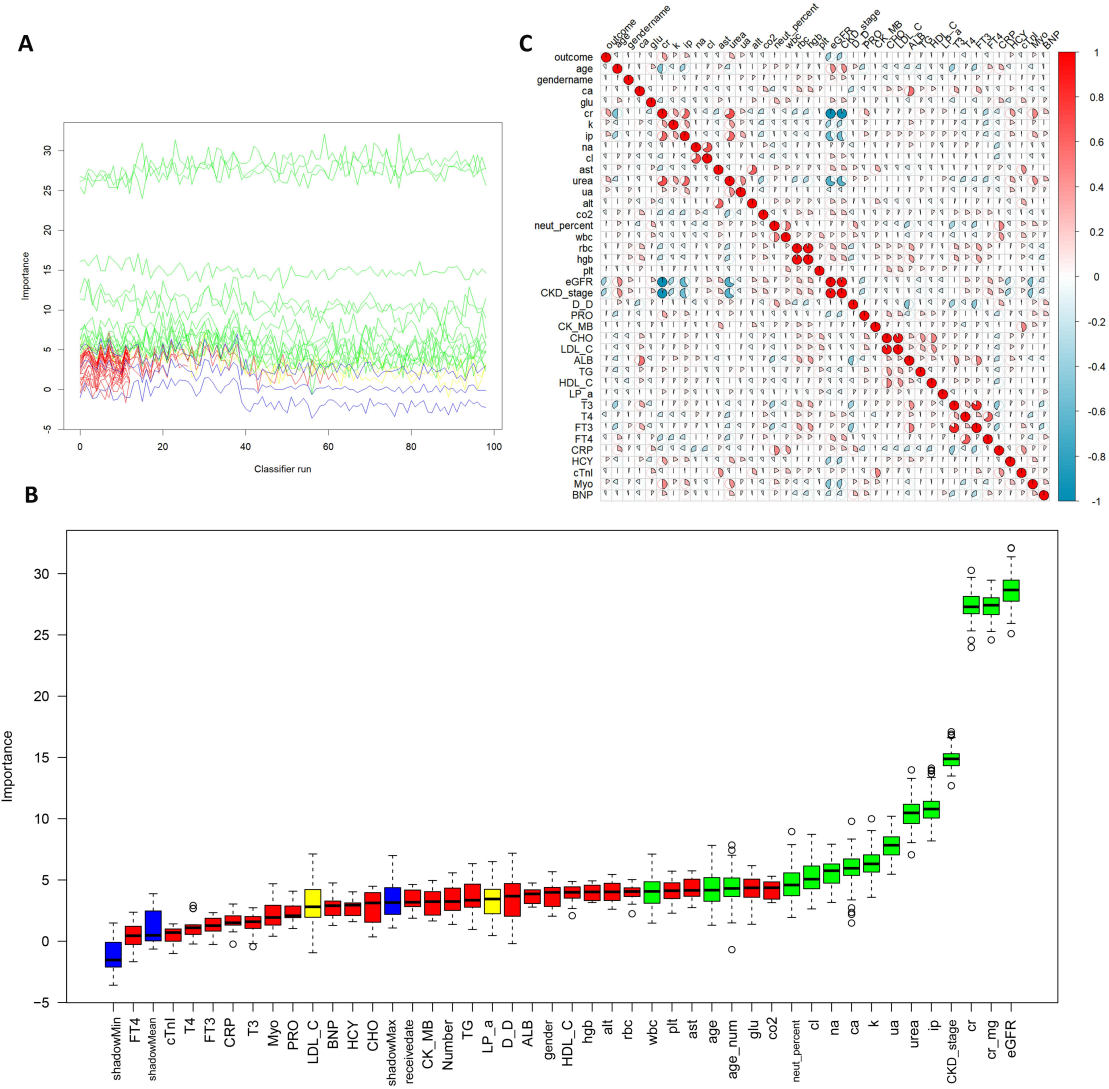
Variables selection based on boruta algorithm. **(A)** Path diagram of changes in feature importance scores during algorithm iterations. **(B)** The ranking of the importance of each clinical variable. **(C)** The Spearman correlation coefficients between the response outcome and the variables.

Variables selection utilizing the Boruta algorithm is depicted in Figure 2, where a total of 41 variables were initially considered. The path diagram illustrating the fluctuation of feature importance scores throughout the algorithm's iterations demonstrated that after 100 iterations, the importance of each variable set remained stable (Figure 2A). Among these, age, gender, calcium (Ca), glucose (Glu), creatinine (Cr), estimated glomerular filtration rate (eGFR), potassium (K), inorganic phosphorus (iP), sodium (Na), chloride (Cl), aspartate aminotransferase (AST), urea, uric acid (UA), alanine aminotransferase (ALT), carbon dioxide (CO2), and neutrophil percentage (Neut_percent), along with white blood cell (WBC), red blood cell (RBC), hemoglobin (HGB), and platelet (PLT) counts, totaling 20 variables, ranked in the top half and were significantly higher than their corresponding shadow features (Figure 2B). The Spearman correlation coefficients between the response outcome and the variables were presented in Figure 2C. The color intensity and size of the ellipse correspond to the magnitude of the correlation coefficient, with darker shades and larger sizes indicating a stronger association. The correlation analysis revealed significant multicollinearity between creatinine (Cr) and estimated glomerular filtration rate (eGFR). Consequently, eGFR was excluded, and Cr was retained due to its clinical directness and consistent performance in subsequent algorithms. The remaining 19 variables (after this exclusion) all exhibited some degree of correlation with the response outcome.

**Figure 3:**
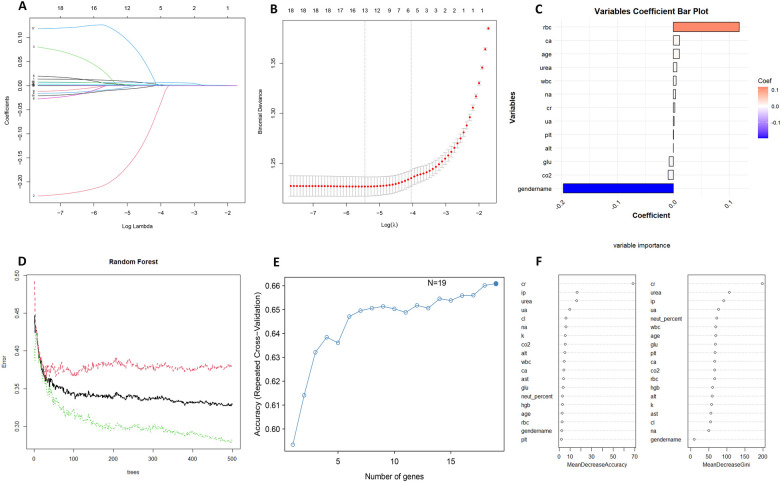
Variable selection based on LASSO and random forest. **(A)** Coefficient profiles of variables across different log(*λ*) values. The vertical dashed line indicates the optimal penalty (lambda.1se) selected by cross-validation. **(B)** Cross-validation curve for LASSO tuning. The optimal *λ* (right dashed line) was chosen within one standard error of the minimum deviance (left dashed line) to promote model sparsity. **(C)** Final coefficients of the 13 variables retained at the chosen *λ*. The bar plot shows the magnitude and direction of each variable's association with the favorable outcome under integrative medicine treatment exposure. **(D)** Random Forest error rate vs. the number of trees. The black line represents the overall Out-of-Bag (OOB) error, demonstrating model convergence. **(E)** Random Forest prediction accuracy via repeated cross-validation vs. the number of variables. The peak accuracy was achieved at 19 variables, indicating the optimal point for computational efficiency. **(F)** Random Forest variable importance plot, ranked by Mean Decrease Accuracy and Mean Decrease Gini. A higher value indicates greater importance for classification.

**Figure 4:**
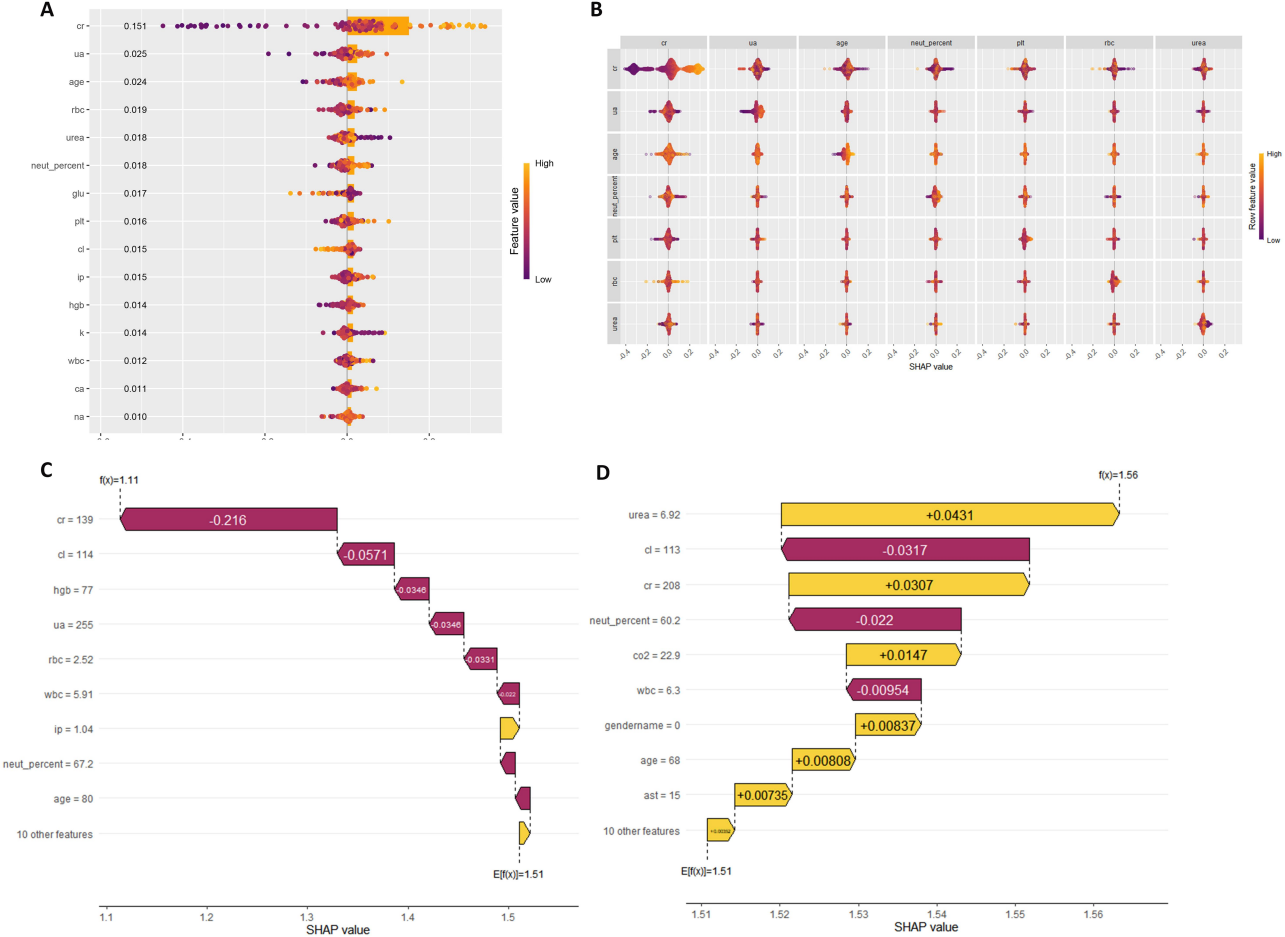
XGBoost-SHAP model analysis. **(A)** Mean absolute SHAP values for the top 15 features, representing their global importance in the model. Creatinine (cr) is identified as the most influential predictor, with a SHAP value approximately 30 times greater than that of sodium (na), which ranks fifteenth. **(B)** Scatterplots of SHAP values for the top 7 features against each other, visualizing their interaction effects and potential correlations. The dispersed point clouds indicate no strong linear correlations between the SHAP values of these top features, suggesting minimal interaction in the model's decision-making process for this cohort. **(C)** Force analysis plot for an individual prediction classified as a non-responder. Features such as high creatinine (cr) and urea push the base model value (E[f**(x)**] = 1.51) to the right, increasing the prediction score towards the non-responder outcome, while features like red blood cell count (rbc) exert a counteracting force. **(D)** Force analysis plot for an individual prediction classified as a responder. In contrast to **(C)**, features like lower chloride (cl) and the individual's gender (gendername) are the primary drivers pushing the prediction score to the left, below the base value, leading to the responder classification.

They were subsequently subjected to LASSO and Random Forest (RF) models, as shown in Figure 3. The LASSO model yielded 13 variables: age, gendername, calcium (Ca), glucose (Glu), creatinine (Cr), sodium (Na), urea, uric acid (UA), alanine aminotransferase (ALT), carbon dioxide (CO2), white blood cell (WBC), red blood cell (RBC), and platelet (PLT) counts. Among these, gendername had the highest weight at approximately 0.1967 and was negatively correlated with the response outcome. Following this, red blood cell (RBC) count had a weight of 0.117 and was positively correlated with the response outcome (Figures 3A–C). The RF model retained all variables, and the model's predictive efficiency was highest when all variables were included. The top three variables were creatinine (Cr), inorganic phosphorus (iP), and urea (Figures 3D–F). The XGBoost-SHAP model identified 15 variables as depicted in Figure 4. The SHAP values for the selected variables indicated that the top three were creatinine (Cr), uric acid (UA), and age (Figure 4A). The SHAP correlation analysis among the top seven features ranked by mean absolute SHAP value showed no significant associations, indicating no evident multicollinearity among these key predictors in the final model (Figure 4B). The distribution of SHAP values for typical responders and non-responders revealed that creatinine (Cr), chloride (Cl), and urea had higher score weights (Figures 4C,D).

### Construction and methodological evaluation of response predictive models

**Figure 5:**
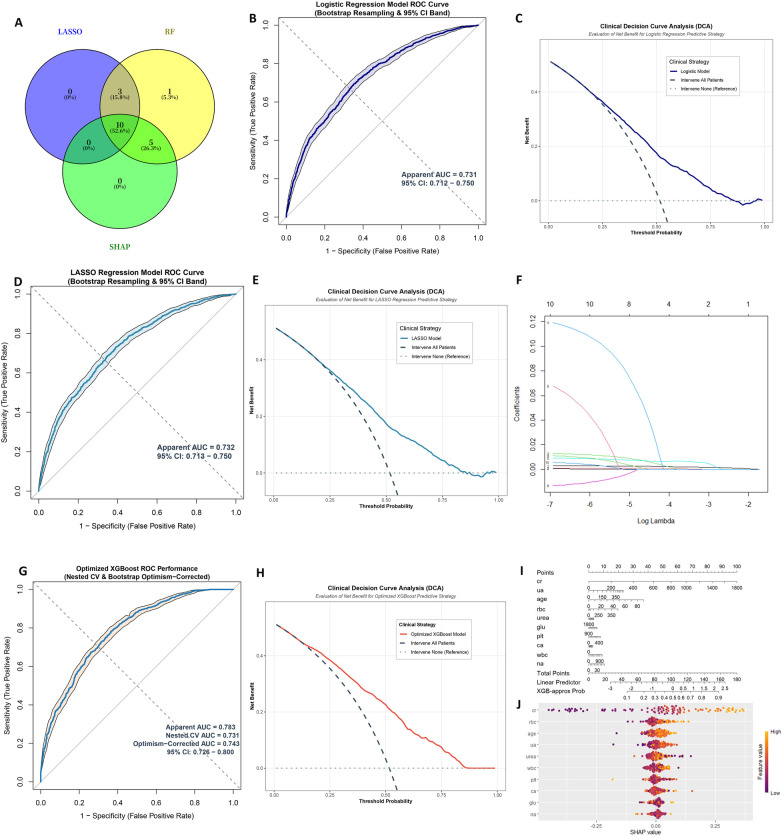
Methodological pipeline for core variable extraction and robust predictive model establishment. **(A)** Venn diagram illustrating the intersection of candidate variables continuously screened by LASSO, Random Forest (RF), and XGBoost-SHAP algorithms, where the central overlapping sector defines the final 10 consensus-selected robust predictors. **(B)** ROC curve of the baseline Logistic Regression model computed with bootstrap resampling, featuring a shaded 95% confidence interval (CI) band and an apparent AUC of 0.731. **(C)** Decision curve analysis (DCA) of the baseline Logistic Regression model evaluating clinical net benefit across the continuum of high-risk threshold probabilities. **(D)** ROC curve of the LASSO Regression model integrated with bootstrap resampling and a 95% CI band, yielding an apparent AUC of 0.732. **(E)** Decision curve analysis (DCA) of the LASSO Regression model, demonstrating its relative clinical utility at various decision thresholds. **(F)** LASSO coefficient profile plot illustrating the dynamic shrinkage paths of predictors as a function of the log-transformed tuning parameter (Log Lambda). **(G)** Advanced ROC performance profile of the optimized XGBoost model,concurrently displaying the Apparent AUC (0.783), the rigorous Nested Cross-Validation (Nested CV) AUC (0.731), and the Bootstrap Optimism-Corrected AUC (0.743) bounded by a 95% CI range (0.726–0.800). **(H)** Decision curve analysis (DCA) plot for the optimized XGBoost model, confirming its superior clinical net benefit structure over traditional strategies. **(I)** Approximate user-friendly nomogram derived via the logistic link, mapping restricted cubic splines (RCS) and remaining linear covariates for individual risk visualization. **(J)** Global SHAP beeswarm summary plot for the XGBoost architecture, sorting the 10 core features by their absolute mean SHAP values to elucidate both the magnitude and directional impact of each indicator on individual predictions.

The construction of predictive models for IMT response was shown in Figure 5. The intersection of variables output by the three algorithms yielded ten clinically relevant variables (Figure 5A), which are creatinine (cr), uric acid (ua), age, red blood cell count (rbc), urea, glucose (glu), platelet count (plt), calcium (ca), white blood cell count (wbc), and sodium (Na). Three parallel classifiers were subsequently evaluated based on these 10 consensus variables. In the training dataset, the optimized XGBoost architecture demonstrated diagnostic superiority over the baseline Logistic Regression and LASSO frameworks across all clinical performance dimensions (Table 2). Imbued with a self-adaptive Youden's Index to optimize the clinical decision cutoff, the XGBoost model achieved a balanced configuration, yielding a True Positive Rate (TPR) of 0.78, a True Negative Rate (TNR) of 0.64, a global Accuracy (ACC) of 0.71, and an F1 score of 0.74, markedly outperforming the linear counterparts.

**Table 2:** Performance of response forecasting models in the training dataset.

Performance metric	Logistic regression	LASSO	XGboost
TPR	0.69	0.73	0.78
TNR	0.65	0.61	0.64
PPV	0.68	0.67	0.70
NPV	0.66	0.68	0.73
ACC	0.67	0.67	0.71
F1 score	0.69	0.70	0.74

TPR, true positive rate; TNR, true negative rate; PPV, positive predictive value; NPV, negative predictive value; ACC, accuracy.

The advanced ROC performance profile of the optimized XGBoost framework concurrently displayed an apparent AUC of 0.783, a rigorous 5-fold Nested Cross-Validation (Nested CV) AUC of 0.731, and a Bootstrap Optimism-Corrected AUC of 0.743 bounded by a tight 95% confidence interval (CI) of 0.726–0.800 (Figure 5G), surpassing the apparent AUCs of the Logistic model (AUC = 0.731; Figure 5B) and the LASSO model (AUC = 0.732; Figure 5D). Furthermore, Decision Curve Analysis (DCA) mathematically confirmed that the optimized XGBoost model generated superior clinical net benefit structures across the entire continuum of high-risk threshold probabilities compared with traditional linear strategies (Figure 5H vs. Figures 5C,E). For bedside utility, a user-friendly nomogram integration mapped the log-linked non-linear predictors (Figure 5I), and the global SHAP beeswarm plots corroborated the supreme predictive weight of serum creatinine in the final ensemble (Figure 5J).

### Algorithmic robustness, calibration, and sensitivity profiling

**Figure 6:**
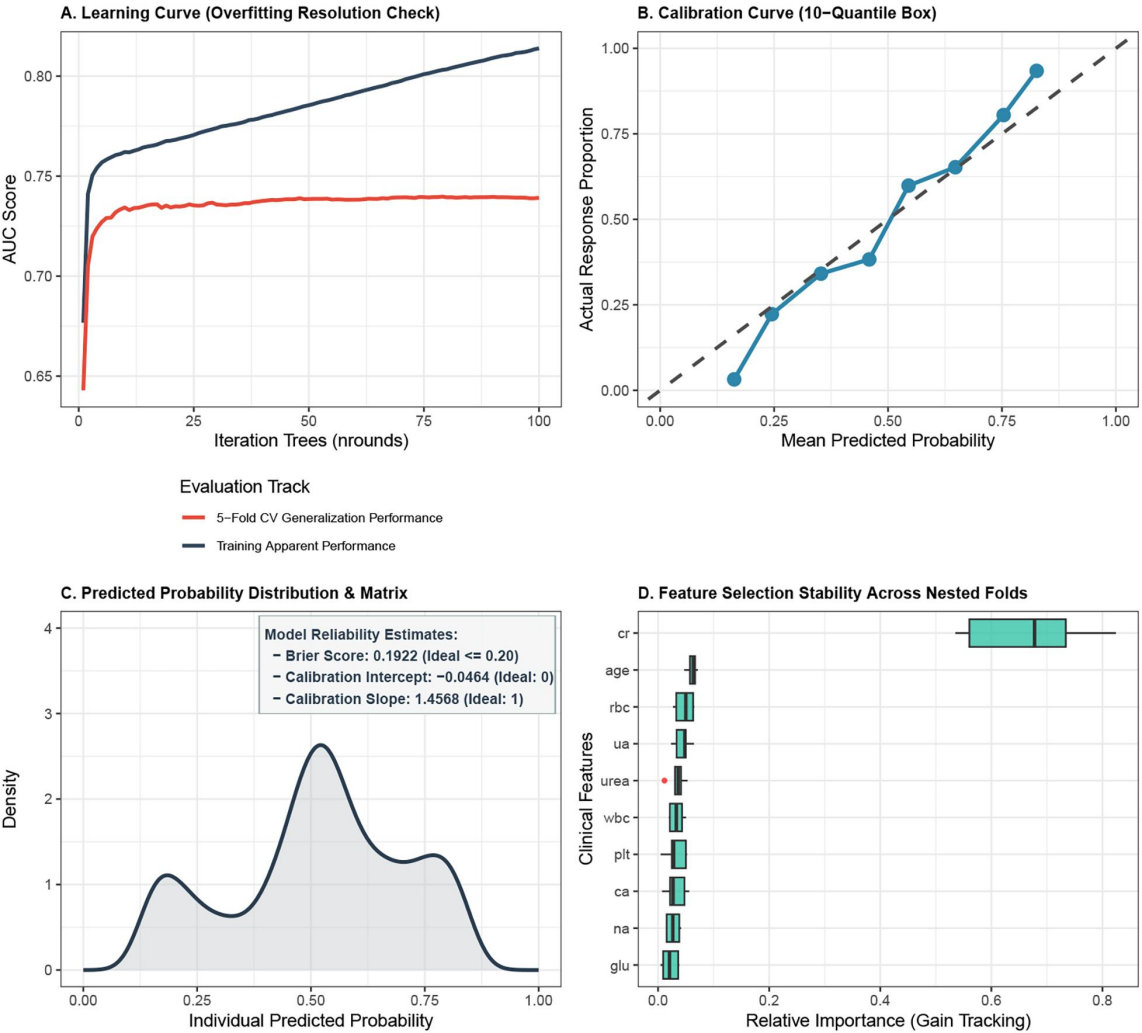
Robustness validation, calibration matrices, and feature stability profiling of the optimized XGBoost framework. **(A)** Learning curves tracking the dynamic convergence of training apparent performance (blue line) and 5-fold cross-validation generalization performance (red line) across progressive decision tree iterations (nrounds), suggesting the mitigation of overfitting risk and the achievement of architectural equilibrium. **(B)** Model calibration curve structured via 10-quantile classification mapping mean predicted probabilities against actual response proportions, where the alignment of the empirical line with the 45-degree reference dashed line demonstrates acceptable agreement between predicted risks and observed clinical outcomes. **(C)** Individual predicted probability distribution density plot paired with key model reliability estimates, explicitly illustrating a well-calibrated Brier score of 0.1922 (Ideal ≤ 0.20), a near-zero calibration intercept of −0.0464 (Ideal: 0), and a calibration slope of 1.4568 (Ideal: 1).While the deviation of the slope from the ideal baseline of 1 indicates a localized under-refinement within extreme risk ranges, care should be taken when interpreting predictions at the tail ends. **(D)** Feature selection stability analysis across nested validation folds, sorting predictors by their relative gain tracking importance and demonstrating the tight, reproducible distribution bounds of critical clinical indicators (such as cr and age) under empirical data perturbations.

To evaluate the mathematical equilibrium of the optimized XGBoost model, rigorous convergence and calibration assessments were executed (Figure 6). Learning curves tracking the dynamic convergence of training apparent performance and 5-fold cross-validation generalization performance across progressive decision-tree iterations reduced the likelihood of confounding (Figure 6A).The 10-quantile classification mapping demonstrated acceptable global calibration, showing reasonable agreement between mean predicted response probabilities and empirically observed clinical event proportions (Figure 6B). This was further verified by the individual distribution density profile, yielding a finely tuned Brier score of 0.1922 (Ideal ≤ 0.20), a near-zero calibration intercept of −0.0464, and a calibration slope of 1.4568 (Figure 6C). The calibration slope departed from the ideal benchmark of 1.0. This suggests a localized under-refinement.These boundary behaviors warrant cautious interpretation. Additionally, feature selection stability analysis across nested cross-validation folds suggested that critical indicators, notably Cr and age, maintained reproducible gain-tracking distribution bounds under empirical data perturbations (Figure 6D).

**Figure 7:**
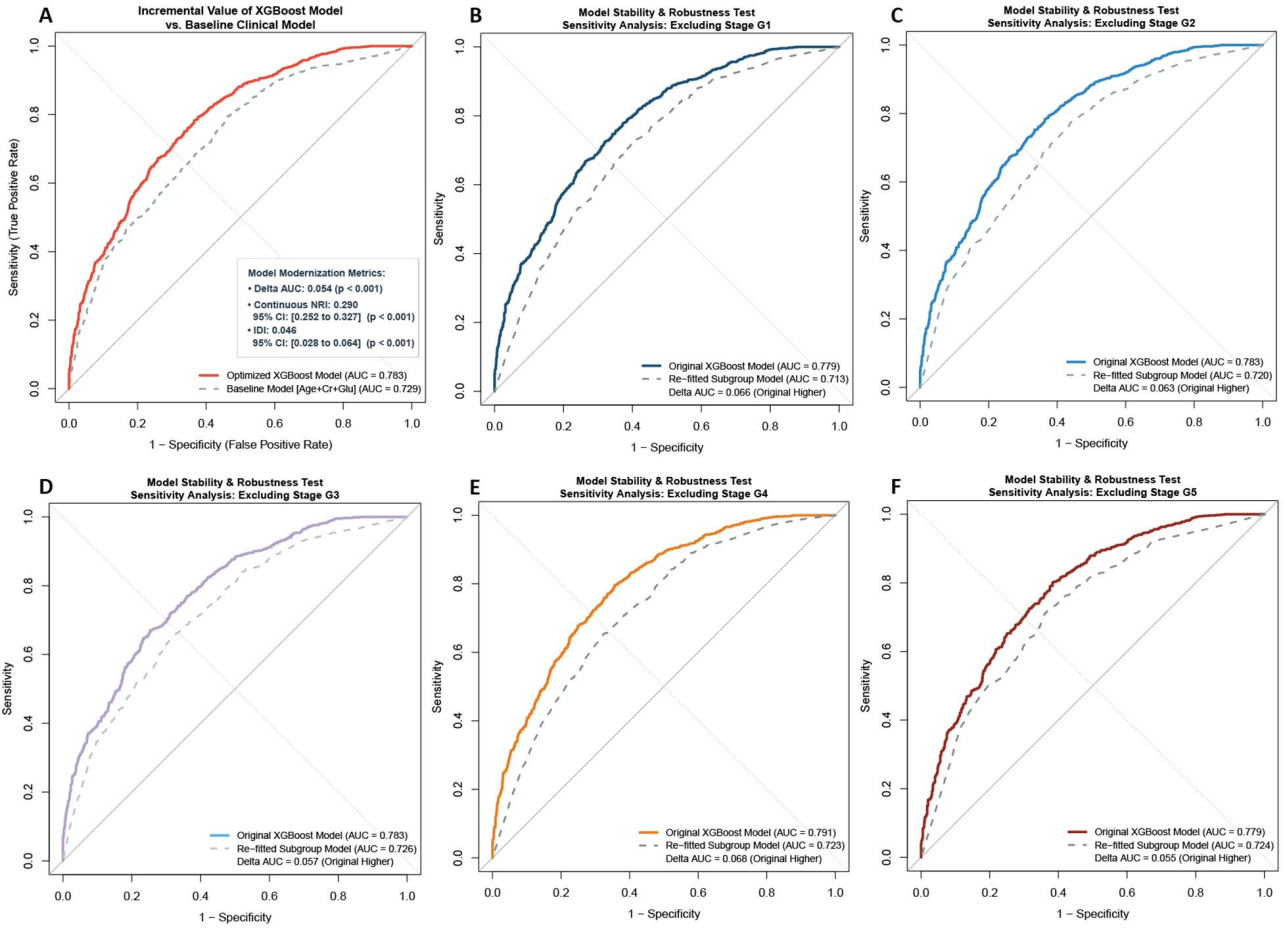
Incremental value evaluation and stage-by-stage sensitivity analysis of the optimized XGBoost framework. **(A)** ROC curve comparison validating the clinical incremental value of the Optimized XGBoost model (apparent AUC = 0.783) against the Baseline Clinical Model (apparent AUC = 0.729). The embedded metric evaluation panel illustrates statistically significant reclassification and discrimination increments, quantified by a Delta AUC of 0.054 (*p* < 0.001), a continuous Net Reclassification Index (NRI) of 0.290 (*p* < 0.001), and an Integrated Discrimination Improvement (IDI) of 0.046 (*p* < 0.001). **(B–F)** Iterative sensitivity analysis curves validating algorithmic stability and robustness across distinct clinical subgroups by sequentially excluding diabetic kidney disease (DKD) patients at specific stages: **(B)** excluding Stage G1, **(C)** excluding Stage G2, **(D)** excluding Stage G3, **(E)** excluding Stage G4, and **(F)** excluding Stage G5. Across all subpopulation perturbations, the performance divergence (Delta AUC) between the original architecture and the re-fitted subgroup models remains bounded within a narrow, negligible interval (0.055–0.068). The minimal divergence observed upon excluding Stage G5 (Delta AUC = 0.055) suggests that the framework's predictive capacity is not merely an artifact of baseline severity; however, it cannot completely rule out subtle confounding effects tied to disease staging.

To comprehensively validate clinical incremental value, the optimized XGBoost model was cross-evaluated against a baseline clinical model (comprising Age + Cr + Glu) (Figure 7A). The optimized model yielded a meaningful reclassification and discrimination gain, characterized by a Delta AUC of 0.054 (*p* < 0.001), a continuous Net Reclassification Index (NRI) of 0.290 (*p* < 0.001), and an Integrated Discrimination Improvement (IDI) of 0.046 (*p* < 0.001).Iterative subpopulation sensitivity analysis curves further assessed algorithmic stability by sequentially excluding patients from specific DKD stages (G1 through G5; Figures 7B–F). Across all subpopulation perturbations, the performance divergence between the original framework and the re-fitted subgroup models remained strictly bounded within a narrow, negligible interval of 0.055–0.068. Crucially, the minimal divergence occurred upon excluding stage G5 patients (Delta AUC = 0.055). Supplementary Figuer 2 showed the Receiver Operating Characteristic Curves Stratified by DKD Stage. The area under the curve (AUC) was 0.821 (95% CI: 0.772–0.871) for Stage G1, 0.789 (95% CI: 0.729–0.849) for Stage G2, 0.781 (95% CI: 0.744–0.818) for Stage G3, 0.752 (95% CI: 0.713–0.791) for Stage G4, and 0.789 (95% CI: 0.762–0.815) for Stage G5. This analysis demonstrated acceptable and consistent discriminative performance across separate homogeneous severity strata, which substantially reduces, but does not entirely exclude, the possibility of residual confounding driven by baseline disease severity.

### Internal validation and external cohort verification

**Figure 8:**
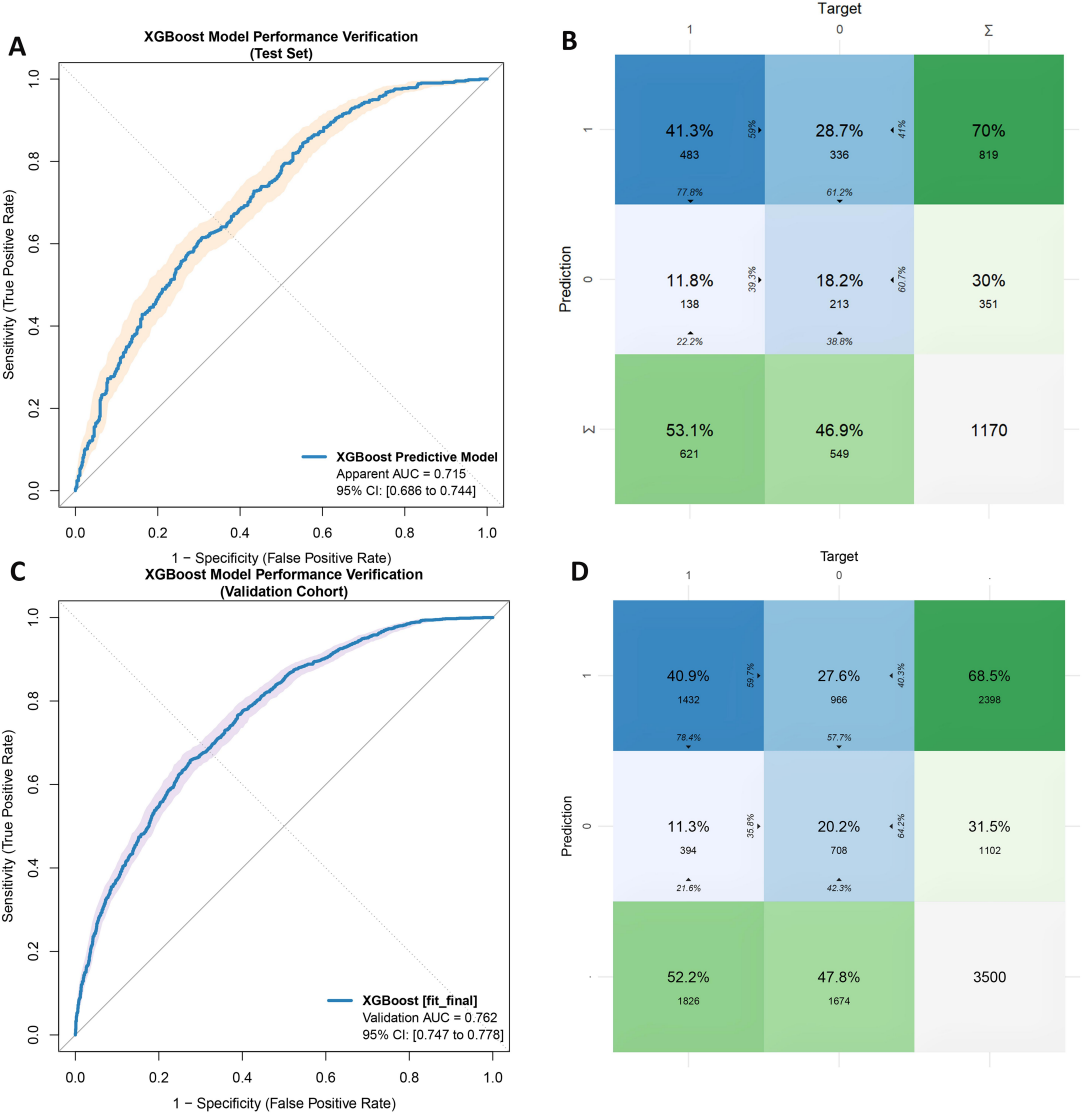
Internal validation and external cohort verification of the optimized XGBoost framework. **(A)** ROC performance verification curve of the optimized XGBoost framework in the independent test set, displaying an Apparent AUC of 0.715 bounded by a shaded 95% confidence interval (CI) range of 0.686–0.744. **(B)** Confusion matrix and derived performance metrics on the internal test set. The model demonstrated a high Sensitivity of 0.77, correctly identifying 77% of actual responders. **(C)** ROC curve of the XGBoost model on the external temporal validation set (2014–2018 cohort), demonstrating a robust validation AUC of 0.762 with a tight 95% CI band spanning 0.747–0.778. **(D)** Confusion matrix and derived performance metrics on the external validation set. The model maintained a similarly high Sensitivity (0.784).

The multi-level generalization capability of the final model was validated using independent internal and external patient series (Figure 8). Within the internal test set (*n* = 1,170), the XGBoost framework sustained a meaningful discrimination performance, achieving an accptable AUC of 0.715 bounded by a shaded 95% confidence interval (CI) range of 0.686–0.744 (Figure 8A). The corresponding two-dimensional confusion matrix and its derived operational performance metrics further substantiated the model's clinical utility; notably, the framework demonstrated the Sensitivity (True Positive Rate, TPR) of 0.77, correctly identifying 77% of the actual therapeutic responders within the internal test population (Figure 8B). Furthermore, external temporal generalization was verified using a temporal validation cohort from 2014 to 2018 (*n* = 3,500; Figures 8C,D). The model architecture had the AUC of 0.762 with 95% CI band spanning 0.747–0.778 (Figure 8C). The external cohort confusion matrix metrics supported the robustness of the model, retaining an optimal Sensitivity (TPR) of 0.784 (Figure 8D).

## Discussion

This study constructed and validated an explainable prediction model for favorable outcome under integrative medicine treatment exposure in adult patients with DKD through a retrospective cohort study. The research process included: screening 7,400 DKD patients from the electronic health records of China-Japan Friendship Hospital between 2010 and 2018; randomly dividing 3,900 patients from 2010 to 2014 into a training set and an internal test set at a 7:3 ratio, with 3,500 patients from 2014 to 2018 serving as an temporal validation cohort; performing feature selection using the Boruta algorithm, LASSO, Random Forest, and XGBoost-SHAP, ultimately identifying 10 core variables such as creatinine, uric acid, and age; constructing three models (logistic regression, LASSO, and XGBoost) based on these variables, evaluating performance through ROC curves, AUC values, and decision curve analysis, and found that the XGBoost model performed optimally with model decisions explained via SHAP values; and finally deploying the model as an interactive web application. Results showed that the model had an AUC of 0.715 in the test set and 0.762 in the validation set, demonstrating acceptable predictive efficacy in distinguishing responders from non-responders to integrative medicine treatment. The web interface is intended for use by nephrologists or endocrinologists at the point of care. It requires no specialised informatics skills: the clinician uploads or types the 10 laboratory values, clicks “Predict”, and receives an instantaneous probability. Patients themselves do not operate the interface, thereby avoiding variability introduced by differences in digital literacy.It should be noted that although the global reliability configuration suggests the model's potential utility for clinical risk stratification, the noticeable departure of the calibration slope (1.4568) from the ideal benchmark of 1.0 requires a cautious interpretation, as it indicates the model may under- or over-estimate the true likelihood of favorable outcomes at the validation extremes. In addition, despite the implementation of various sensitivity analyses,the possibility of residual confounding driven by baseline disease severity was not excluded entirely.

Previous studies on DKD prediction models have mostly focused on disease progression (e.g., eGFR decline or end-stage kidney disease occurrence) within a Western medicine treatment context. For example, researchers in Xuzhou Central Hospital, China have extracted the electronic health records of 12,190 T2DM patients with 3-year follow-ups and the dataset was divided into a training and testing dataset in a 4:1 ratio. Using the importance ranking in the random forest package, the variables of age, urinary albumin-to-creatinine ratio, serum cystatin C, estimated glomerular filtration rate, and neutrophil percentage were selected as the predictors for DKD onset. They provided a powerful tools for early DKD risk prediction (33). Furthermore, some researchers collected 268 observation values from two tertiary hospitals in Lanzhou, China. These included demographic information, basic medical history, as well as routine laboratory tests such as blood routine, common biochemical tests, and urine routine. They respectively applied traditional statistical methods and machine learning methods to establish models, and determined the best prediction model for DKD (34). Although traditional regression models or machine learning models based on clinical indicators (such as proteinuria, blood glucose, and blood pressure) can predict the risk of DKD progression, They lack individualized assessment of favorable outcome under integrative medicine treatment exposure. Some studies have attempted to incorporate genetic or protein markers to improve prediction accuracy (35–37), but their clinical application is limited due to small sample sizes or high detection costs of markers. Compared with this study, few models have addressed response prediction for the intervention of integrative medicine and paid attention to model interpretability. This study tried to construct a model for predicting response to integrative medicine in DKD patients, enhancing interpretability through SHAP values and nomograms, and filling the gap in traditional models for supporting individualized treatment decisions.

The model generated by this study provided a practical tool for identifying the dominant population of integrated medicine. Currently, the identification of this clinical dominant population was still in the exploratory stage. Some scholars have defined this population as patients who, under the guidance of evidence-based medicine, receive standardized treatment and, after concurrent Traditional Chinese Medicine (TCM), have better outcomes in terms of survival, duration, tumor shrinkage, and quality of life compared to those receiving standard modern medical treatment (38). Although numerous retrospective studies based on the Taiwan health insurance plan have attempted to analyze the characteristics of the population benefiting from TCM, they usually only performed simple linear regression statistics or only distinguished simple subgroups such as gender and age (39–41), so the resulting population profiles were not detailed. There are also studies focusing on the clinical characteristics and syndrome elements of cancer patients receiving integrated medicine treatment (42, 43), but few studies have focused on the clinical characteristics of chronic disease patients, especially those with diabetic kidney disease (DKD), who benefit from it.

This study has certain limitations: first, the samples were derived from a single center. Given the single-center nature of this research, the model's findings reflect institutional practices and specific patient demographics, meaning its general applicability to other healthcare settings cannot be assumed. Multicenter and geographically independent external validation remains strictly necessary before routine clinical implementation; second, the model only included routine clinical indicators and not multi-omics data such as genes and metabolomes, potentially missing potential predictive factors; third, we were unable to reliably retrieve or standardize individual-level longitudinal compliance data for ACEI/ARB exposure to perform accurate statistical stratification or algorithmic adjustment. Given the well-established landmark role of renin-angiotensin-aldosterone system (RAAS) inhibition in reducing albuminuria and slowing long-term renal failure progression in diabetic kidney disease, the lack of granular, time-varying medication titration logs presents an inherent clinical limitation. More broadly, patient treatment adherence and long-term compliance to complex therapeutic regimens remain potential unmeasured confounders that are inherently difficult to capture with granular precision within retrospective electronic health records. Given that individual-level variance in adherence directly influences clinical outcomes and renal trajectories, the inability to mathematically adjust for compliance patterns means that residual confounding cannot be entirely eliminated.In addition, It is plausible that observed variations are influenced by institutional referral patterns or variations in patient treatment adherence in late-stage cohorts. As our current registry lacks direct quantified metrics for these behavioral variables, such explanations remain strictly hypothetical. These observed stratifications should be interpreted with caution and require dedicated prospective validation.

Consequently, although our multi-stage cross-validation and subgroup diagnostics support the statistical robustness of the model, residual confounding related to unmeasured medication exposure or individual-level treatment compliance variance cannot be entirely excluded, and our predictive outputs must be interpreted within these retrospective boundaries.

Future research can be optimized in the following aspects: conducting multi-center, prospective cohort studies across geographically diverse healthcare networks to expand sample representativeness and rigorously evaluate the model's external transportability; evaluating the integration of additional emerging and conventional biomarkers, specifically embedding tubular injury markers [such as Neutrophil Gelatinase-Associated Lipocalin [NGAL] or Kidney Injury Molecule-1 [KIM-1]] and alternative filtration markers (such as Cystatin C), to explore potential increments in individualized risk discrimination and net reclassification (NRI/IDI) in diabetic kidney disease; refining model stratification by incorporating patients' TCM syndromes and types of Chinese medicine; developing dynamic prediction models that include changes in indicators during treatment to improve long-term prediction efficacy. The ultimate goal is to establish an interpretable and clinically supportive risk-stratification tool, thereby assisting clinicians in optimizing individual-level integrative medicine treatment strategies for DKD within compatible clinical boundaries.

## Conclusions

We developed and internally validated an explainable model to predict likelihood of favorable outcome under integrative medicine treatment (IMT) exposure in adult DKD patients via a machine learning approach, which used common clinical and biochemical features that are readily available in routine clinical practice.

## Data Availability

The original contributions presented in the study are included in the article/Supplementary Material, further inquiries can be directed to the corresponding authors.
