# Supplementary material for: Identification and validation of an explainable prediction model of favorable outcome under integrative medicine treatment exposure in DKD adult patients: a retrospective cohort study
Source: Front Digit Health. 2026 Jul 16;8:1803468. doi: 10.3389/fdgth.2026.1803468 (PMC13422240; doi:10.3389/fdgth.2026.1803468)
Supplement: Supplementary file 1 [file Datasheet1.docx]

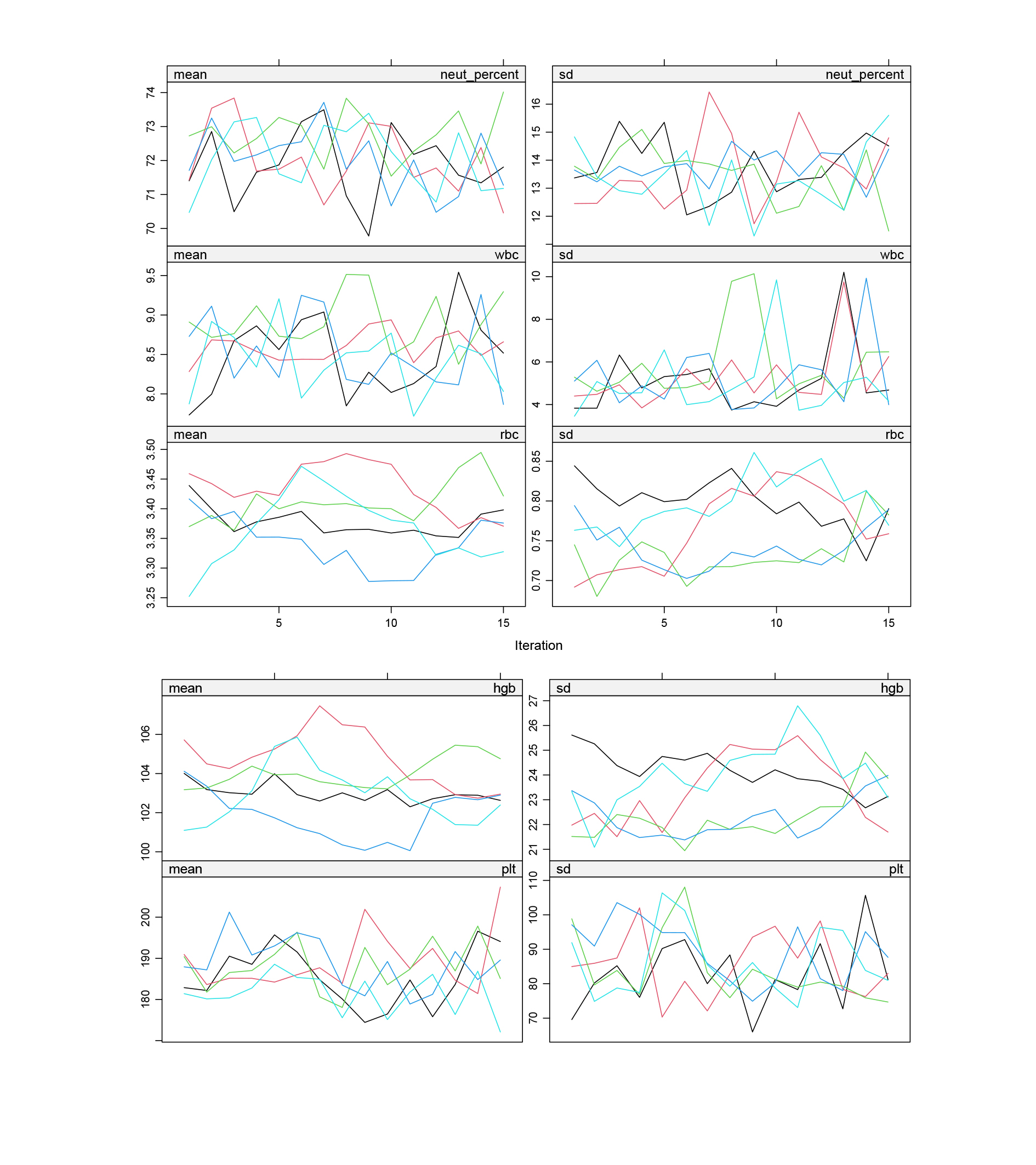


**Supplementary fig.1** Post-imputation convergence diagnostics of the MICE algorithm.Trace plots

illustrating the mathematical convergence of the Multivariate Imputation by Chained Equations

(MICE) framework. The left panels display the mean trajectories, and the right panels display the

standard deviation trajectories across 15 iterations for five independent imputation chains

(colored lines), covering neutrophil percentage (neut_percent), white blood cell count (wbc), red

blood cell count (rbc), hemoglobin (hgb), and platelet count (plt). The stable, stochastic

interweaving of multi-chain trajectories without systematic drift or divergence confirms adequate

convergence and verifies that the imputed values preserved the natural clinical variance of the

original cohort.


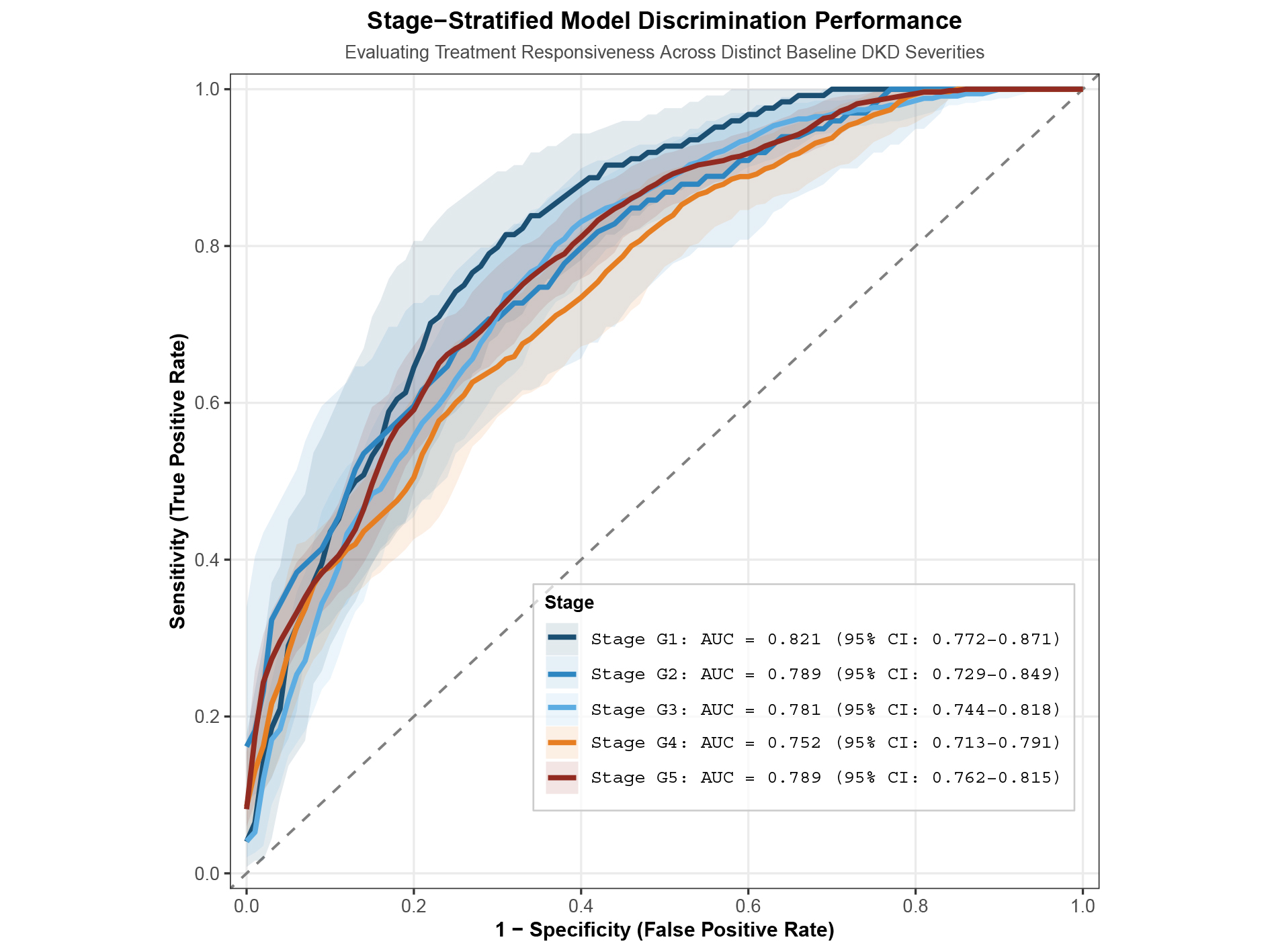


**Supplementary fig.2** Receiver Operating Characteristic Curves Stratified by DKD Stage. The area under the curve (AUC) was 0.821 (95% CI: 0.772–0.871) for Stage G1, 0.789 (95% CI: 0.729–0.849) for Stage G2, 0.781 (95% CI: 0.744–0.818) for Stage G3, 0.752 (95% CI: 0.713–0.791) for Stage G4, and 0.789 (95% CI: 0.762–0.815) for Stage G5, suggesting stable treatment responsiveness prediction irrespective of baseline disease severity.

Supplementary Table 1 the details of optimized hyperparameters

| Hyperparameter | Description | Optimized Value |
| --- | --- | --- |
| n_estimators | Number of boosting rounds | 150 |
| max_depth | Maximum tree depth for base learners | 3 (Shallow trees to prevent overfitting) |
| learning_rate (eta) | Step size shrinkage used in update to prevent overfitting | 0.05 |
| subsample | Subsample ratio of the training instances | 0.8 |
| colsample_bytree | Subsample ratio of columns when constructing each tree | 0.7 |
| reg_alpha (L1) | L1 regularization term on weights | 0.1 |
| reg_lambda (L2) | L2 regularization term on weights | 1.5 |
| scale_pos_weight | Balancing of positive and negative weights | Adjusted based on baseline response ratio |
